# Prognostic value of the expression of cancer stem cell-related markers CD133 and CD44 in hepatocellular carcinoma: From patients to patient-derived tumor xenograft models

**DOI:** 10.18632/oncotarget.10164

**Published:** 2016-06-18

**Authors:** Qihong Zhao, Heng Zhou, Qifei Liu, Ye Cao, Gang Wang, Anla Hu, Liang Ruan, Sufang Wang, Qingli Bo, Wenjun Chen, Chuanlai Hu, Dexiang Xu, Fangbiao Tao, Jiyu Cao, Yongsheng Ge, Zongfan Yu, Li Li, Hua Wang

**Affiliations:** ^1^ Department of Food and Nutrition Hygiene, School of Public Health, Anhui Medical University, Hefei, China; ^2^ School of Pharmacy, Anhui Medical University, Hefei, China; ^3^ Department of Oncology, The First Affiliated Hospital of Anhui Medical University, Hefei, China; ^4^ Department of Oncology, Affiliated Provincial Hospital of Anhui Medical University, Hefei, China; ^5^ Department of General Surgery, Affiliated Provincial Hospital of Anhui Medical University, Hefei, China; ^6^ Department of General Surgery, The Second Affiliated Hospital of Anhui Medical University, Hefei, China; ^7^ Institute for Liver Disease, Anhui Medical University, Hefei, China

**Keywords:** prognostic value, cancer stem cell markers, hepatocellular carcinoma, patient-derived tumor xenograft models

## Abstract

High expression of cancer stem cell (CSC) markers is related to poor prognosis of patients with hepatocellular carcinoma (HCC). However, the expression of these markers in patient-derived xenograft (PDX) models and the relationship of the expression levels of these markers between HCC patients and their PDX models at subsequent low passages are unclear. To investigate the prognostic impact of putative CSC markers in patients with HCC and in related PDX models, the expression of CD133, CD90, CD44, ALDH1, CK7, CK19, OCT4, SOX2, vimentin, nestin, CD13 and EpCam were assessed by quantitative reverse transcription-PCR (qRT-PCR) and then were validated using immunohistochemistry in tumor or peritumoral tissues from patients and tumor tissues from PDX models. Cumulative survival analysis of the patients and animals was conducted using the Kaplan-Meier method and the log-rank test. Only the expression levels of CD133 and CD44 were higher in tumor tissues than in the peritumoral tissues of HCC patients by qRT-PCR. High consistency of the prognostic value of the expression of CD133/CD44 was observed in HCC patients and the PDX models. High expression levels of CD133 and CD44 were positively related to the poor prognosis of HCC patients and to that in the PDX models. PDX HCC models in the present study have been suggested to be predictive of disease outcome, which could shed light on personalized medicine and the mechanisms of CSC marker expression on prognosis.

## INTRODUCTION

Accumulating evidence has shown that cancer cells might be hierarchically organized, similar to tissue-specific stem cells [1]. Further, a subset of cancer cells possesses stem cell properties, which are called cancer stem cells (CSCs), might be responsible for long-term renewal potential and maintenance [2–4]. More importantly, CSCs could survive after surgical treatment and chemotherapy due to their quiescent status [5] or high-efficiency detoxifying [6]. Hepatocellular carcinoma (HCC) remains one of the most prevalent cancer types in past decades despite great advances in novel therapies and the development of anti-cancer drugs [7]. The incidence and mortality rates, along with the recurrence rate, have been increasing, both in China [8, 9] and in other countries worldwide [7, 10].

CSC markers have been identified in several types of cancers for CSC isolation and characterization. Considering that different CSC markers have demonstrated different behaviors on CSCs, such as stemness maintenance and epithelial-mesenchymal transition (EMT) [11], we chose twelve markers: CD133, CD90, CD44, ALDH1, CK7, CK19, OCT4, SOX2, vimentin, nestin, CD13 and EpCam. These markers have remarkable features. CD133 was exhibited by proliferative cells in multiple organs and was identified as a CSC marker in many types of tumors [12]; CD90 is a glycosylphosphatidylinositol-anchored glycoprotein expressed in many types of CSCs [13]; CD44, which evolved into multiple signaling transductions as a cell surface molecule, has been used as a CSC marker [14]; aldehyde dehydrogenase-1 (ALDH-1) is highly expressed in various tumorigenic cells and has shown great potential as a CSC marker for the isolation and identification of CSC cells from multiple types of tumors [15, 16]; cytokeratin 7 and 19 (CK7 and CK19) are components of the cancer cell cytoskeleton and are responsible for prediction of the early recurrence and prognosis [17, 18]; octamer-binding transcription factor 4 (OCT4) and SOX2, are key transcription factors that regulate pluripotency [19, 20]; vimentin and nestin are EMT-related markers with the ability to develop into multiple cell lineages [21, 22]; CD13 is a novel liver CSC marker and a candidate therapeutic target [23]; and epithelial cell adhesion molecule (EpCam), also called CD326, could serve as an early biomarker and novel prognostic marker of HCC [24]. However, these markers are expressed not only in tumor tissues but also in the peritumoral/stromal tissues [25, 26]. Therefore, it is necessary to detect the expression levels of these markers in the tumor area and the peritumoral area of the same tissue, which could provide new understanding of the putative CSCs within the microenvironment, as well as the peritumoral-supportive cells.

Patient-derived xenograft (PDX) models are established by the transfer of fresh tumors from surgery into immunodeficient mice on the subcutaneous side. When the tumors grew too large for the first generation mice, we passaged the tumors over to the next generation of mice. This type of model has been attracted increasing attention due to its advantages, such as maintenance of the cellular complexity and architecture for the donor, which mimics the tumor microenvironment at subsequent low generations [27]. These models could overcome the obstacles in the studies of CSCs [2] and could facilitate the application of personalized medicine.

The purpose of the present study was first to investigate the mRNA expression of CD133, CD90, CD44, ALDH 1, CK7, CK19, OCT4, SOX2, vimentin, nestin, CD13 and EpCam in tumor and peritumoral tissues from HCC patients and then to validate the protein expression of these markers with significant differences in mRNA levels in the tumor and peritumoral tissues of HCC patients. In PDX models, tissues were grouped by the expression levels of the original clinical HCC tissues, and the mRNA expression of these CSC markers was subsequently tested in newly grown tumor tissues in mice to explore prognostic role of these markers both in HCC patients and in the HCC PDX models to elucidate the relationship between them. Using quantitative reverse transcription-PCR (qRT-PCR) and immunohistochemistry staining in 74 primary HCC patients with full demographic/clinicopathological data and follow-up, we analyzed the effects of these markers on the survival time of the patients, and then we validated these results in the PDX models using the same methods.

## RESULTS

### The mRNA expression of CSC-related markers in HCC tissues

The relative expression of CD133, CD90, CD44, ALDH1, CK7, CK19, OCT4, SOX2, vimentin, nestin, CD13 and EpCam from 74 tumor tissues and the peritumoral tissues was detected and analyzed.

**Figure 1:**
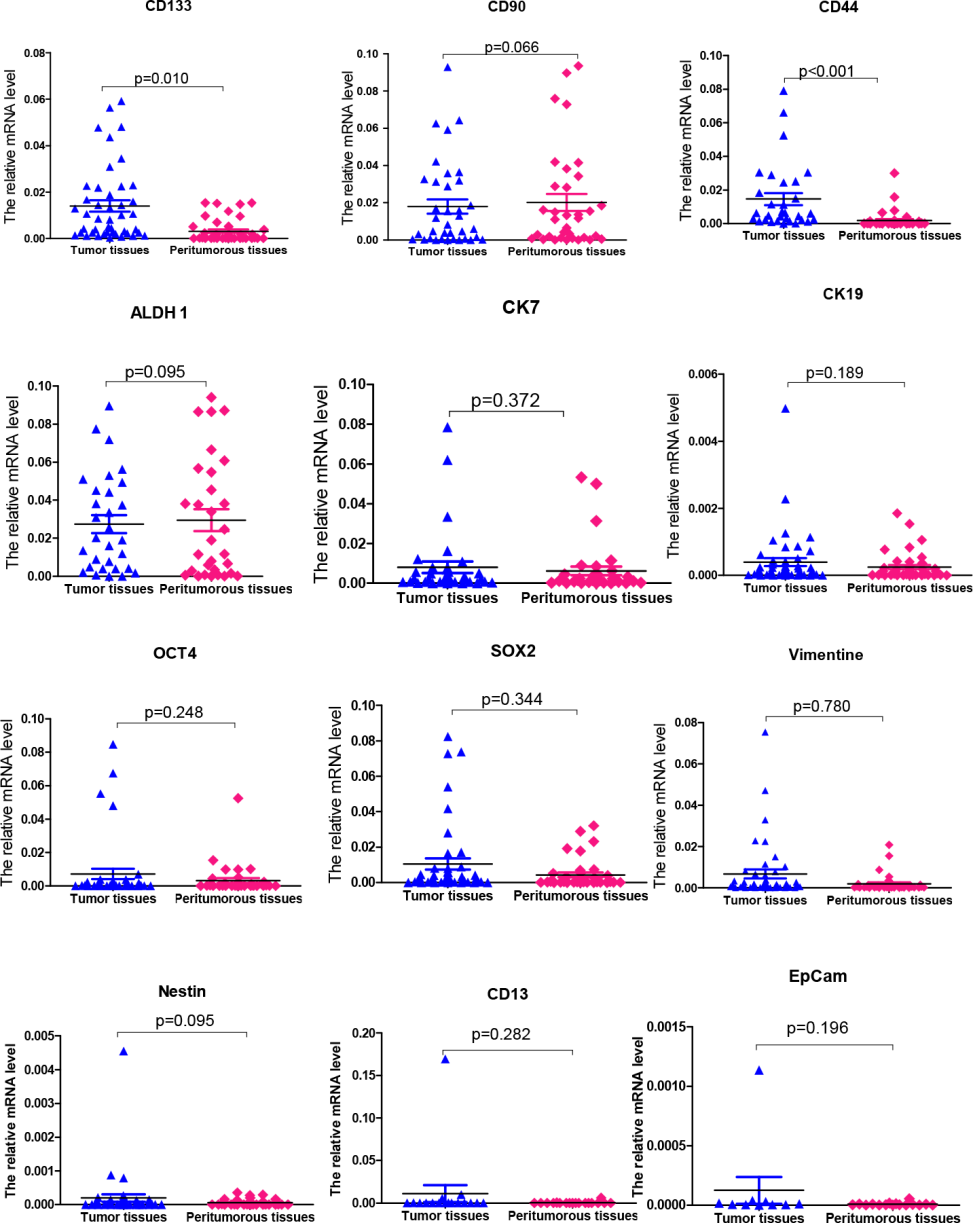
The mRNA expression levels of 12 putative CSC-related markers in tumor tissues and peritumoral tissues of patients with HCC

We observed that the expression of 10 of 12 CSC-related markers (CD90, ALDH1, CK7, CK19, OCT4, SOX2, vimentin, nestin, CD13 and EpCam) was not significantly different between the tumor tissues and peritumoral tissues. However, the expression of CD133 and CD44 had statistically significant differences between the two groups (*p* = 0.010 and *p* < 0.001, respectively). The results are shown in Figure 1.

### Protein expression of CD133 and CD44 in the tumor and peritumoral tissues of HCC patients

**Figure 2:**
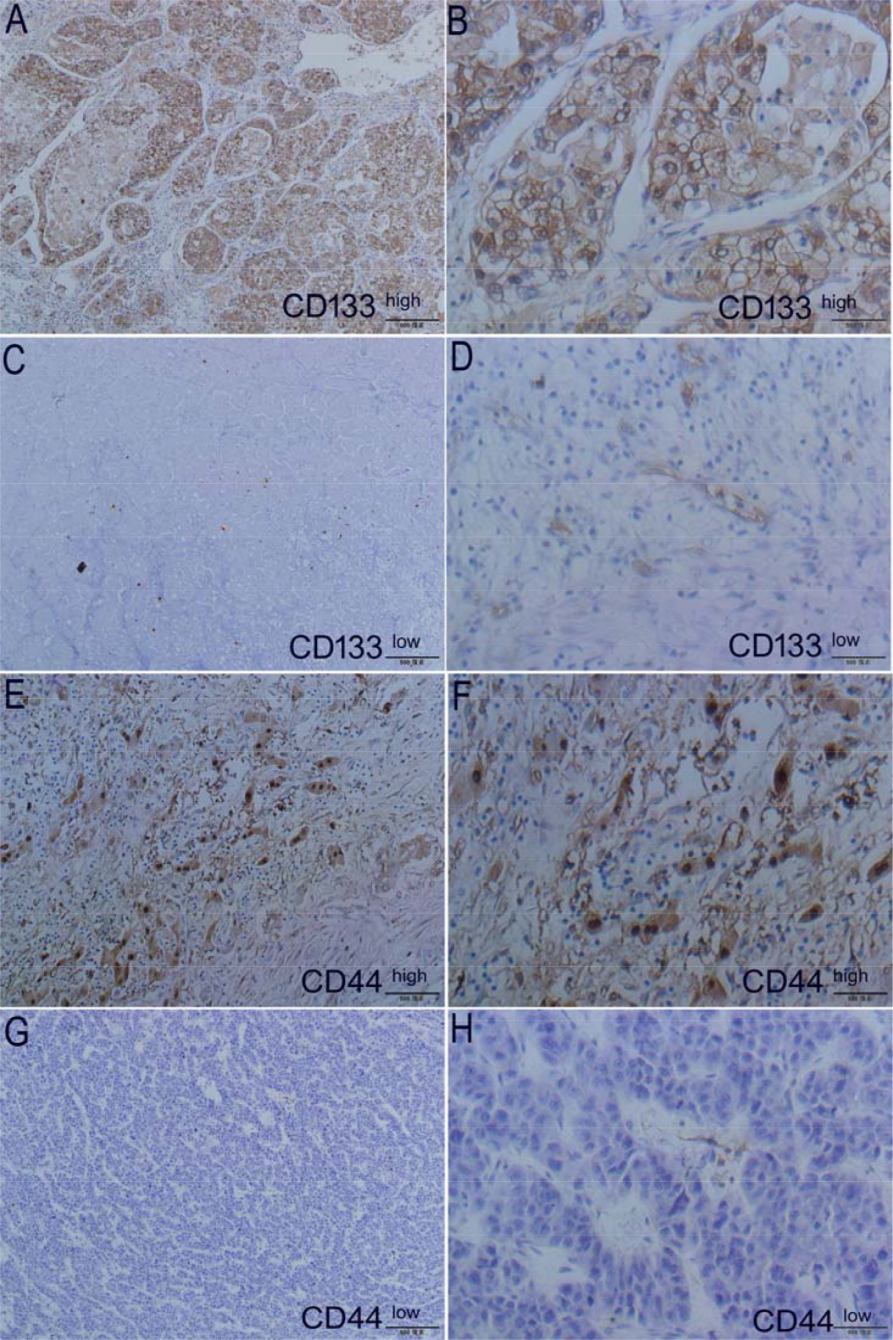
Expression of markers CD133 and CD44 in tumors using HE staining (left panel) and IHC (right panel) from patients Representative case 1 HE staining (**A**) and its high expression of CD133 (**B**) *vs* case 2 HE staining (**C**) and its low expression of CD133 (**D**); Representative case 3 HE staining (**E**) and its high expression of CD44 (**F**) *vs* case 4 HE staining (**G**) and its low expression of CD44 (**H**). Original magnification for all pictures is 400×.

The expression of CD133 and CD44 in tumor and peritumoral tissues was detected by IHC. The respective expression of CD133 and CD44 is shown in Figure 2. The histological sections of the same tissue after HE staining are displayed in Supplementary Figure S1. After scoring, we found that 55.41% of CD44 cases and 58.11% of CD133 cases were highly expressed. We also found CD133 and CD44 simultaneously in 27 of the 74 cases (36.49%) that had medium/high co-expression. Detailed quantification of the samples stained by the scoring system and the IHC scores for CD44 and CD133 is shown in Supplementary Figure S2.

### Prognostic significance of CSC markers (CD133 and CD44) and clinicopathological characteristics

**Figure 3:**
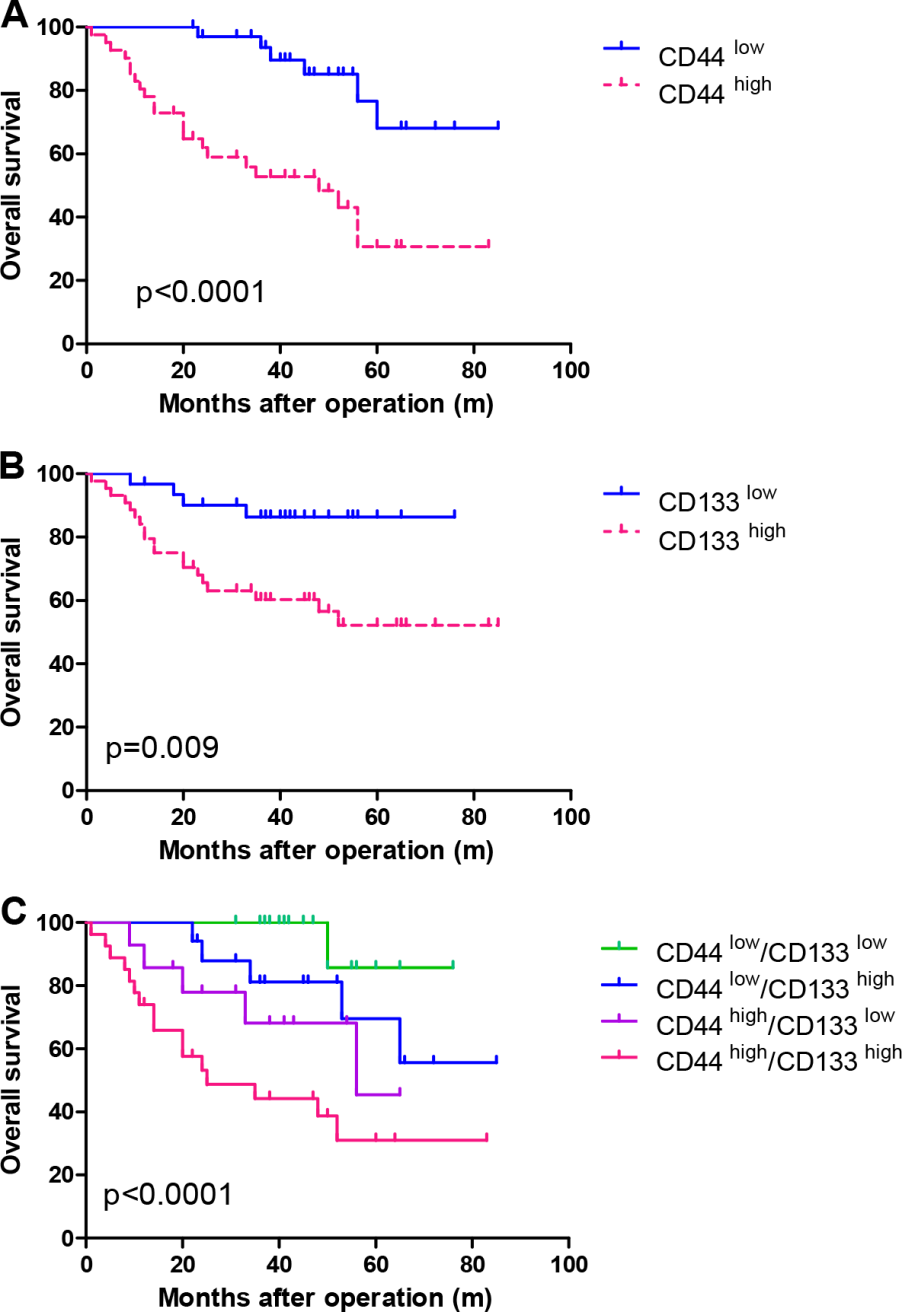
Kaplan-Meier survival curves showing survival time differences in patients with (A) high or low CD44 expression, (B) high or low CD133 expression and (C) co-expression of CD133 and CD44 using immunohistochemistry staining

Over the 7-year follow-up, the mean survival time of the HCC patients with low CD44 expression was 73.20 ± 4.17 months, while that in HCC patients with high CD44 expression was 44.84 ± 5.20 months by survival analysis. There was a statistically significant difference between the two groups (*p* < 0.001). The results are shown in Figure 3A.

Regarding the expression of CD133, we found that the mean survival time of HCC patients in the low expression group was 68.42 ± 3.55 months, compared with 54.36 ± 5.30 months in the high expression group. The statistical difference was also significant between the two groups (*p* = 0.007), as shown in Figure 3B.

Due to co-expression of CD133 and CD44 in some cases, we divided these HCC patients into four groups— CD44^low^/CD133^low^, CD44^low^/CD133^high^, CD44^high^/CD133^low^ and CD44^high^/CD133^high^—to analyze the prognostic effects of these two factors. The mean survival times of these four groups were 72.29 ± 3.44 months, 67.53 ± 6.30 months, 48.55 ± 5.93 months and 40.71 ± 6.51 months, respectively. Significant differences were significant among these four groups (*p* = 0.001). The results are shown in Figure 3C.

In contrast, we analyzed the association of CD133- and CD44-expression with demographic data and clinicopathological features in HCC patients (see Table 1). Only liver cirrhosis had effects on the expression of CD133 (*p* < 0.01).

**Table 1:** Associations of the expression of CD133 and CD44 with the demographic data and clinicopathological characteristics in HCC patients

Variables	Cases	CD133 expression		*P* values	CD44 expression		*P* values
		Low	High		Low	High	
**Age, years**							
< 52	38	15	23	0.084	19	19	0.337
≥ 52	36	16	20		14	22	
**Sex**							
Male	64	24	40	1.000	27	37	0.502
Female	10	7	3		6	4	
**HBsAg**							
Negative	11	10	1	0.444	4	7	0.294
Positive	63	21	42		29	34	
**HBeAg**							
Negative	58	19	39	0.471	27	31	0.979
Positive	16	12	4		6	10	
**Liver cirrhosis**							
No	13	7	6	**0.006**	7	6	0.460
Yes	61	24	37		26	35	
**Tumor differentiation**				1.000			0.552
I–II	55	26	29		23	32	
III–IV	19	5	14		10	9	
**Tumor stage**				0.519			0.945
I–II	44	32	12		20	24	
III–IV	30	23	7		12	18	
**Tumor size**				0.305			0.066
> 5 cm	8	6	2		2	6	
≦ 5 cm	66	49	17		30	36	
**Tumor number**				0.960			0.404
1–2	71	52	19		31	40	
≥ 3	3	3	0		1	2	
**AFP^1^ (ng/ml)**				0.368			0.176
< 40	48	28	20		24	24	
≥ 40	26	3	23		9	17	
**ALT^2^ (U/l)**				0.301			0.243
< 75	32	12	20		10	22	
≥ 75	42	19	23		21	21	
**Child-Pugh**				1.000			1.000
A	70	27	43		31	39	
B	4	4	0		2	2	

1 AFP, a-fetoprotein.

2 ALT, alanine aminotransferase.

### The mRNA expression of CSC-related markers in cancer tissues of PDX models

After establishing the patient-derived tumor xenograft models successfully, we detected the relative mRNA expression of the 12 CSC-related markers in the tumor tissues from the models. Based on the protein expression of CD133 and CD44 in the clinical tumor samples, the patient-derived tumor xenograft models were divided into four groups: high CD133 expression, low CD133 expression, high CD44 expression and low CD44 expression groups.

**Figure 4:**
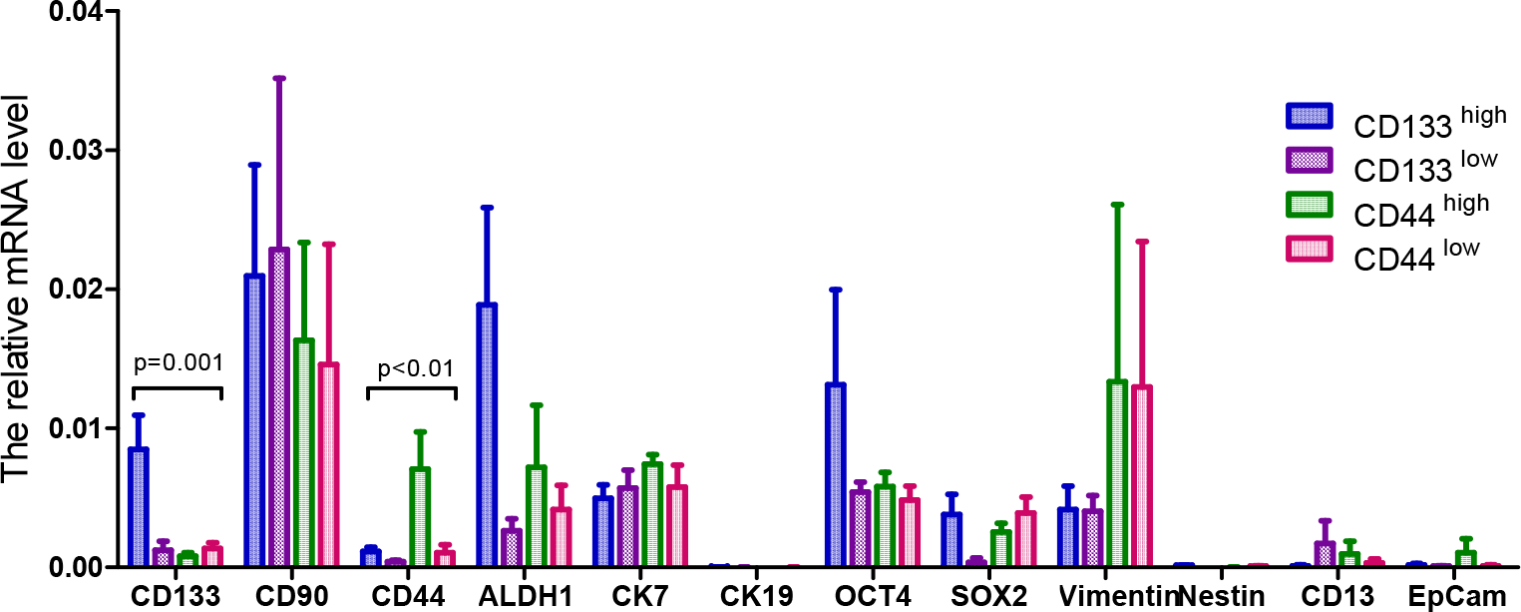
The mRNA expression level of 12 putative CSC-related markers in the tumor tissues from the HCC PDX models

As shown in Figure 4, the relative mRNA expression of the 12 CSC-related markers (CD133, CD90, CD44, ALDH1, CK7, CK19, OCT4, SOX2, vimentin, nestin, CD13 and EpCam) in these four groups was heterogeneous. However, there were statistically significant differences in the CD133 and CD44 relative mRNA expression levels among these four groups (*p* = 0.001 and *p* < 0.01, respectively). The other ten mRNA expressions of the CSC-related markers in this study had no statistically significant differences among the groups.

### Protein expression of CD133 and CD44 in cancer tissues from PDX models

**Figure 5:**
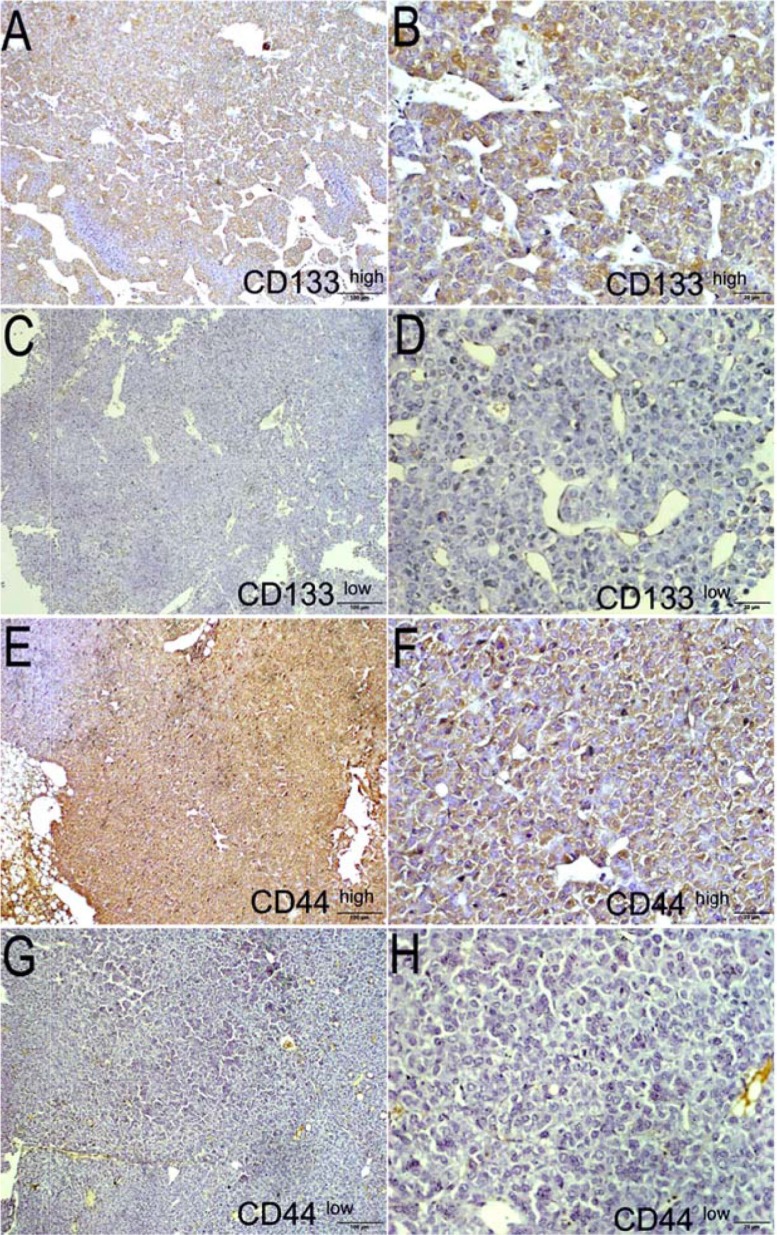
Expression of markers CD133 and CD44 in tumors using HE staining (left panel) and IHC (right panel) from PDX models Representative HE staining (**A**) and its expression of CD133 (**B**) from the CD133 high expression group *vs* HE staining (**C**) and its expression of CD133 (**D**) from the CD133 low expression group; Representative HE staining (**E**) and its expression of CD44 from the CD44 high expression group (**F**) *vs* HE staining (**G**) and its expression of CD44 (**H**) from the CD44 low expression group. Original magnification for all pictures is 400×.

Protein expression of CD133 and CD44 in the cancer tissues of the PDX models was in concordance with that in the clinical tumor tissue samples using IHC assay. In the PDX models established from the clinical tissues with high expression of the proteins CD133 and CD44, we observed that high expression of CD133 and CD44 was inherited (Figure 5A, 5B, 5E and 5F). Correspondingly, low expression of CD133 and CD44 is illustrated in Figure 5C, 5D and Figure 5G, 5H in the tumor tissues from the PDX models, which were established using clinical tissues with low expression of the CD133 and CD44 proteins. Histological sections of each model are presented in Supplementary Figure S3 after HE staining.

In the re-growth-screening process of the tumor cells in immune-deficient mice, it seemed that there was little influence on the expression of some proteins, which could be viewed as good inheritance of this type of model by the clinical tumor samples.

### Prognostic value of CD133 and CD44 in PDX models

**Figure 6:**
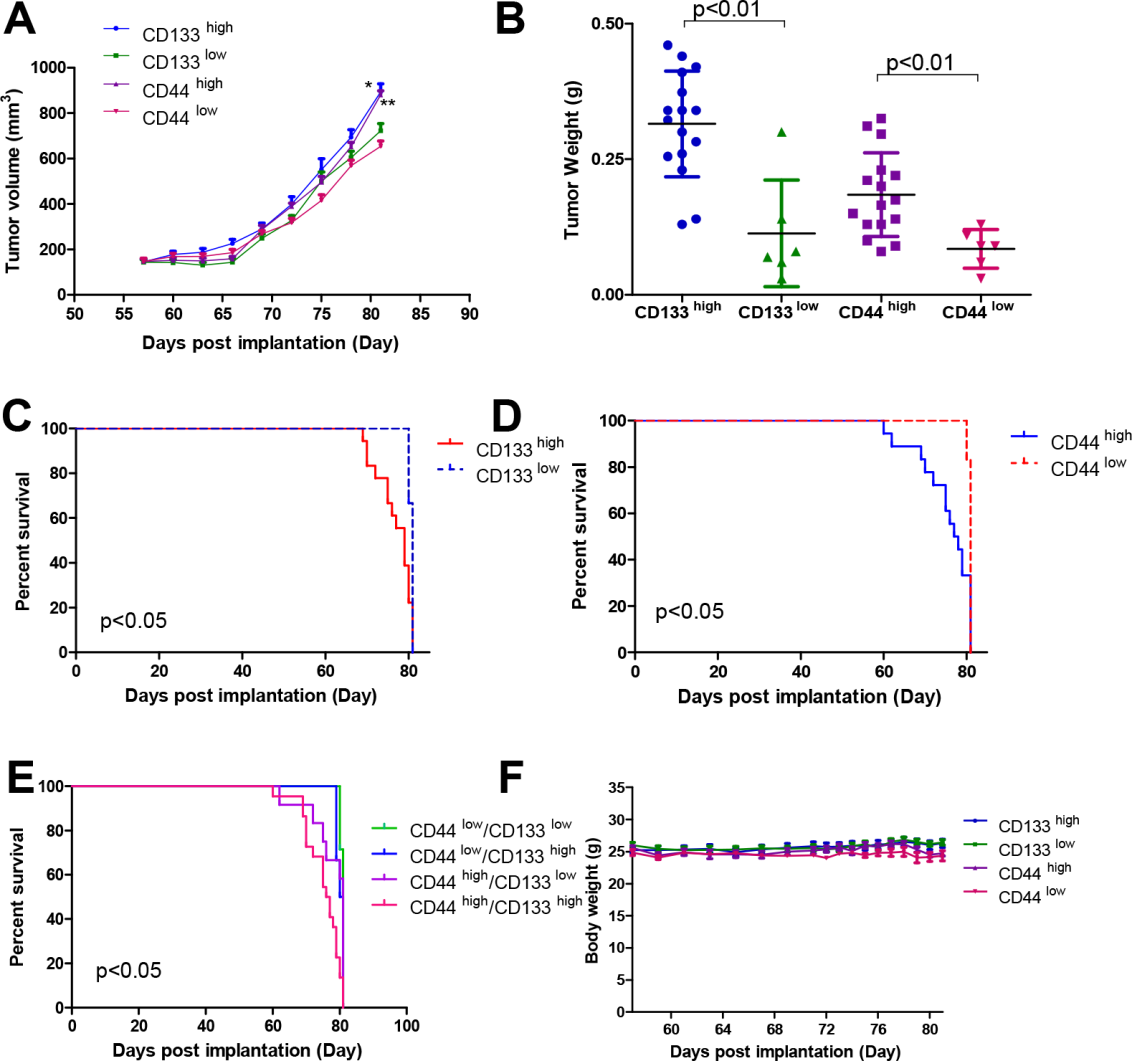
Tumor growth, tumor weight, Kaplan-Meier survival curves and the body weight changes of mice in PDX models (**A**) CD44 illustrated the tumor growth in different groups of PDX models and (**B**) showed the tumor weight at the end point. Survival time differences in (**C**) high or low CD133expression group, (**D**) high or low CD44 expression, (**E**) co-expression of CD133 and CD44 and (**F**) the body weight changes of mice during the whole experiment. *p < 0.05 in high CD133 group *vs* low CD133 group and **p < 0.01 in high CD44 group *vs* low CD44 group.

Before we evaluated the prognostic effects of the expression of CD133 and CD44 in PDX models, we first analyzed the tumor growth of different groups. We observed that the tumors in the CD133 and CD44 high expression groups grew faster than those in their corresponding low expression groups (*p* < 0.05 and *p* < 0.01, respectively) (Figure 6A). At the end point of the experiment, we weighed the tumor weight of each group, and similar results were observed (as shown in Figure 6B).

In the CD44 high/low expression groups of the PDX models, the mean survival time of the high CD44 expression group (*n* = 18) was 78.67 ± 0.71 days after implantation. In the low CD44 expression group (*n* = 6), the mean survival time was 80.83 ± 0.15 days after implantation. There was a statistically significant difference between the two groups (*p* < 0.05). The percentage survival of each group is shown in Figure 6C.

Similar results were found in the CD133 groups. The mean survival time of the high CD133 expression group was 79.78 ± 0.46 days after implantation, but that in the low CD133 expression group was 80.83 ± 0.15 days after implantation. The statistical difference was significant between these two groups (*p* < 0.05). The results are shown in Figure 6D.

In addition to the co-expression of CD133 and CD44 in clinical tumor samples, the co-expression of CD133 and CD44 was also observed in the tumor samples from the PDX models. Based on the co-expression levels of CD133 and CD44, we divided the PDX models into four groups—CD44 low/CD133 low, CD44 low/CD133high, CD44 high/CD133 low and CD44 high/CD133high—to analyze the prognostic effects of these two factors. The percentage survival of these four groups is shown in Figure 6E. The mean survival times of these four groups were 80.86 ± 0.13 days, 78.56 ± 0.79 days, 79.91 ± 0.64 days and 79.24 ± 0.54 days after implantation, respectively. The statistical difference was significant among these four groups (*p* < 0.05). At the same time, the similar body weight fluctuations between groups can be seen in Figure 6F in the same breeding environment. IHC scores for CD133 and CD44 in the PDX models are shown in Supplementary Table S2.

## DISCUSSION

Patient-derived xenograft (PDX) models have been widely applied in translational research and have shown more predictive behavior for clinical outcomes. Precise identification of cancer stem cell (CSC) populations could help to characterize the subtypes of cancer patients and could contribute to personalized therapies.

In the present study, we first evaluated the mRNA expression of 12 CSC-related markers and found the significant differences in the mRNA expression of CD133 and CD44 between clinical HCC tumor tissues and their peritumoral tissues. Then, we tested the protein expression of CD133 and CD44 in clinical HCC tumor tissues and observed that higher expression of each marker (3 or more using IHC scoring system) had a poorer impact on the survival time of patients. Subsequently, we validated these findings in PDX models and found that the tumors established from the clinical HCC tissues with high expression of CD133 and CD44 grew faster than those established from clinical HCC tissues with low expression of CD133 and CD44. The differences were still statistically significant, although there were not large differences in the mean survival time, which might have had their special characteristics regarding the tumor growth of the PDX models. At the same time, the analysis of the survival time of the tumor-bearing mice was consistent with that observed in the clinical patients with the same expression status of these markers.

CD133, a transmembrane glycoprotein, was found in human hematopoietic stem/progenitor cells and was initially considered as a hematopoietic stem cell-specific surface marker. However, subsequent research demonstrated that CD133 was expressed not only in neural stem cells and epidermal stem cells but also in CSCs [28, 29]. Many previous works described that increased expression of CD133 from tumor tissues was correlated with poor prognosis in colorectal cancer [30], ovarian cancer [31], non-small cell lung cancer [32], gastric cancer [33], cholangiocarcinoma [34], and pancreatic cancer [35], in agreement with the results in HCC of this study. CD44 is widely expressed on the cell surface as an adhesion glycoprotein. It also has been studied as a marker of CSCs. Many early works also found that high expression of CD44 from many types of tumor tissues had a worse impact on survival time in patients [36–40]. In contrast, these CSC markers are not always dependable for the whole process of tumor development [2]. Some markers might be lost in the development of the tumors, such as sonic hedgehog (Shh). Shh was expressed on stem cells in the basal urothelium and formed aggressive colonies *in situ*. Subsequently, invasive tumors were generated, but Shh expression was lost within this lesion [41]. Therefore, in this study, we validated the expression of these two markers in PDX models. Further, expression of these markers was observed in the tumors from PDX models consistent with that in the patients. At the same time, we also analyzed that the prognostic values of the co-expression of these two markers in survival time. More shortened survival time was observed in the group with double high expression of CD133/CD44, as well as in the tumor-bearing animals.

Although the average survival time between the high expression groups and low expression groups was close, we could still determine the trends in survival time in two groups by combining the tumor growth rate and the tumor weight at the end of the study. Furthermore, there remained significant differences in these three parameters between the groups.

Regarding demographic data, such as sex and age, we did not find correlations with the expression of CD133/CD44. Although hepatitis B virus (HBV) infection is an important cause of HCC, we did not observe an effect of HBV on the expression of CD133/CD44 using two hepatitis B surface antigens, HBsAg and HBeAg, as indicators in the present study, indicating the non-viral origin of HCC with high expression of these markers [42]. Regarding the clinicopathological characteristics, we observed that liver cirrhosis had effects on CD133 expression (*p* = 0.006) but no effect on CD44 expression in these HCC patients. CD133 expression could be more sensitive than CD44 expression to the composition changes in the process of HCC with liver cirrhosis, which might cause this difference in expression. Tumor differentiation and tumor stage are the basic indicators for evaluating tumor growth and the prognosis of patients [43]. AFP and ALT are also very widely used tumor markers in HCC diagnosis and management. The results of the present study indicated that there was no effect of HCC differentiation, tumor stage, tumor size, tumor number or AFP/ALT levels on CD133/CD44 expression.

Owing to the great heterogeneity of HCC, the combination of several markers can significantly increase the predictive power [44, 45]. We also observed shorter survival time in the group with co-expression of CD133/CD44, both in patients and in the PDX models. The mean tumor weight at the end point also showed that the tumors with high expression of CD133 or CD44 grew faster than those with corresponding low expression of CD133 and CD44. Therefore, the co-expression of several CSC markers might be more predictive of the prognosis of patients at high expression.

In this study, we observed that the mRNA expression of CD133 and CD44 among 12 CSC markers in the tumor tissues of HCC patients was significantly different from that in peritumoral tissues. We also found that the protein expression of CD133 and CD44 was heterogeneous and that their protein expression levels were positively correlated with the survival time of patients. Regarding the other ten CSC markers, we did not detect their protein expression. That no difference was found in mRNA levels does not indicate that there was no difference in the protein level. In this regard, we could discuss this topic in future studies or review previous reports [11, 46, 47]. We focused on the CSC markers found in the mRNA levels to analyze whether these markers play a key role in prognostic prediction for HCC patients. Conversely, we applied PDX models to validate these intriguing findings. This application could compensate for the limitations of our study, such as the loss of information on local recurrence, distant metastasis and post-operative treatment. Our results showed high consistency with this combined system of patients and PDX models. Our findings indicated that high expression of CSC markers, CD133 and CD44, was predictive of poor clinical outcome, while the application of PDX models also had predictive effects on clinical outcomes, as well as in the guidance of personalized medicine.

## MATERIALS AND METHODS

### Patients and tissue samples

Seventy-four patients (64 men and 10 woman) with the mean age of 45.2 ± 16.6 years old (range, 28–72 years) who underwent surgical resection of primary HCC at Anhui Medical University from 2007 to 2013 were recruited with written informed consent before they participated in this study. Ethical approval for the present study was obtained from the ethics committee of Anhui Medical University.

Fresh tumor specimens and their peritumoral tissues (at a distance of at least 30 mm from the tumor edge) were obtained immediately after surgery from all of the patients, according to protocols approved by the ethics committee of Anhui Medical University. All of the data were analyzed anonymously throughout the study. Once the specimens arrived in the lab, each specimen was divided into three parts: the first part was maintained in RNAlater (Life Technology, USA) for qRT-PCR analysis; the second part was fixed with formalin and then embedded in paraffin for TMA analysis; and the third part was used to establish the PDX models. Other important clinical data, including clinicopathological characteristics, from each patient were obtained from their medical records at the same time, and they are summarized in Table 1. Follow-up was terminated on 30 June 2013. Cumulative overall survival was evaluated from the surgery to the last observation.

### Real-time qRT-PCR analysis

To investigate the mRNA levels of CSC-related genes, total RNA from the cancer tissues of HCC patients and PDX models was reverse transcribed using a SuperScript III RT reagent Kit (Invitrogen Life Technologies, Carlsbad, CA, USA). Quantitative real-time PCR was performed using SYBR Green I Master (Roche Diagnostics, Germany) on a LightCycler 480 instrument (Roche Diagnostics, Germany).

Gene expression levels were calculated based on the following equation: 2 ^−ΔCt^ [ΔCt = Ct (Target)-Ct (GAPDH)]. All of the samples were measured in duplicate. The conditions for qRT-PCR were as follows: 5 min at 94°C and then 50 cycles of 94°C for 30 s, 59°C for 30 s, and 72°C for 1 min. Supplementary Table S1 shows the details of the primers.

### Immunohistochemistry (IHC) staining and evaluation

Slides 4 μm in thickness were prepared by a Leica RM2265 rotary microtome (Germany). After deparaffinization in xylene and rehydration in graded alcohols, slides were placed in a 1% (w/v) zinc sulfate antigen retrieval solution and boiled for 30 min in a microwave for antigen retrieval. Following incubation with 3% H_2_O_2_ to quench the endogenous peroxidase, the slides were blocked in 5% BSA for 2 h at room temperature, and then incubation with the primary antibodies CD133 (1:200, BD) and CD44 (1:200, BD) was performed in a moist chamber overnight at 4°C. After being washed twice in PBS, the slides were then incubated for 1 h in the corresponding HRP-conjugated secondary antibody (1:10,000) diluted in 1% BSA. Eventually, the slides were developed with 2, 3-diaminobenzidine tetrahydrochloride for 10 min and were counterstained with hematoxylin for 30 s. The slides were then washed, dehydrated in graded alcohol, and mounted with neutralized gummi.

The expression of these markers was scored based on intensity and percentage of positively stained cells. For the intensity evaluation, a 4-score scale was applied: 0, negative staining; 1 +, weak; 2 +, moderate; and 3 +, strong intensity. The percentage of positive cells was evaluated according to the following criteria: Score 0 (no stained or < 10% stained cells), Score 1 (11–50% stained cells), Score 2 (51–80% stained cells), and Score 3 (> 80% stained cells). The expression patterns were independently evaluated by two pathologists blinded to the clinical outcomes. Therefore, the valid range of scores was 0–6 from the combined density and intensity analyses. For statistical analysis, the scores were further classified into three groups: negative/low expression (0–2), medium expression (3–4) and high expression (5–6) of staining.

### Animals

Eight-week-old female NOD/SCID mice (Beijing Vital River, China) were used for the implantation of the clinical tumor samples. They were housed and maintained in a specific pathogen-free (SPF) facility under standard laboratory conditions (25 ± 2°C, 12:12 h light-dark cycle) and received food and water *ad libitum*. The experiments were conducted in accordance with the ethics committee on animal experimentation and the animal welfare regulations of Anhui Medical University, and the protocol of this study was also approved by ethics committee of Anhui Medical University.

### PDX model establishment

Five female, eight-week-old NOD/SCID mice were used for the implantation of the each of the clinical tumor samples within 8 hours after the surgery. Briefly, approximately 1 mm^3^ of clinical HCC tissue was transplanted into immunodeficient mice on the subcutaneous side, and they propagated in the mice directly. When the tumors grew up to a proper size, the newly grown tumor, at approximately the same volume, was transplanted to next generation. The surplus of the newly grown tumors was cryo-preserved for implantation after thawing at 37°C.

Subsequently, eight PDX models were chosen from our model list established from 2007 to 2013, including three CD133-positive cases and one CD133-negative case, in addition to three CD44-positive cases and one CD44-negative case. The generation of mice transplanted with clinical tumor tissues was regarded as F0. They were transferred to next generation as F1, and the measurement of the expression of CSC-related markers was conducted in F2.

Tumor growth, assessed using Vernier calipers, and the mouse body weights were measured twice per week. The tumor volume was calculated using the formula V = width^2^ × length × 0.52. Survival studies were conducted according to the ethical guidelines in which the humane endpoint was the criterion to sacrifice each mouse.

### Statistical analysis

Statistical analysis was performed using SPSS software (version 10.0for Windows). The χ^2^ test, Fisher's exact probability and one-way ANOVA were used to determine the differences between the groups. The threshold for statistical significance was *p* < 0.05. All of the survival analyses were performed using the Kaplan-Meier method and the log-rank test.

## SUPPLEMENTARY MATERIALS FIGURES AND TABLES


