# Supplementary material for: Prognostic value of the expression of cancer stem cell-related markers CD133 and CD44 in hepatocellular carcinoma: From patients to patient-derived tumor xenograft models
Source: Oncotarget. 2016 Jun 18;7(30):47431–43. doi: 10.18632/oncotarget.10164 (PMC5216952; doi:10.18632/oncotarget.10164)
Supplement: Supplementary file 1 [file oncotarget-07-47431-s001.pdf]

Prognostic value of the expression of cancer stem cell-related markers CD133 and CD44 in hepatocellular carcinoma: From patients to patient-derived tumor xenograft models

Supplementary Materials

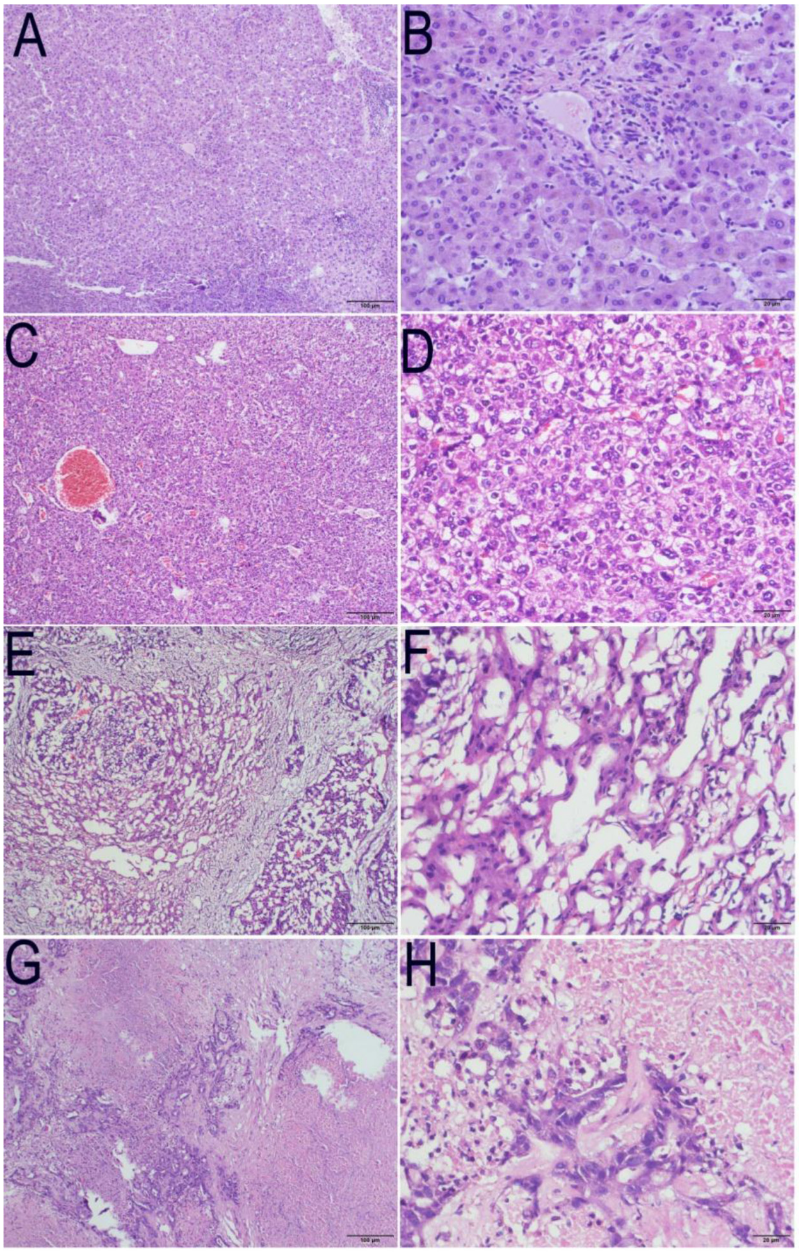

Supplementary Figure S1: Low magnification (left panel, 100×) and high magnification (right panel, 400×) using HE staining from the IHC staining cases of the patients. Representative case 1 HE staining (**A–B**) and its high expression of CD133 in Fig 2 vs case 2 HE staining (**C–D**) and its low expression of CD133 in Fig 2; Representative case 3 HE staining (**E–F**) and its high expression of CD44 in Figure 2 vs case 4 HE staining (**G–H**) and its low expression of CD44 in Figure 2 of the text.

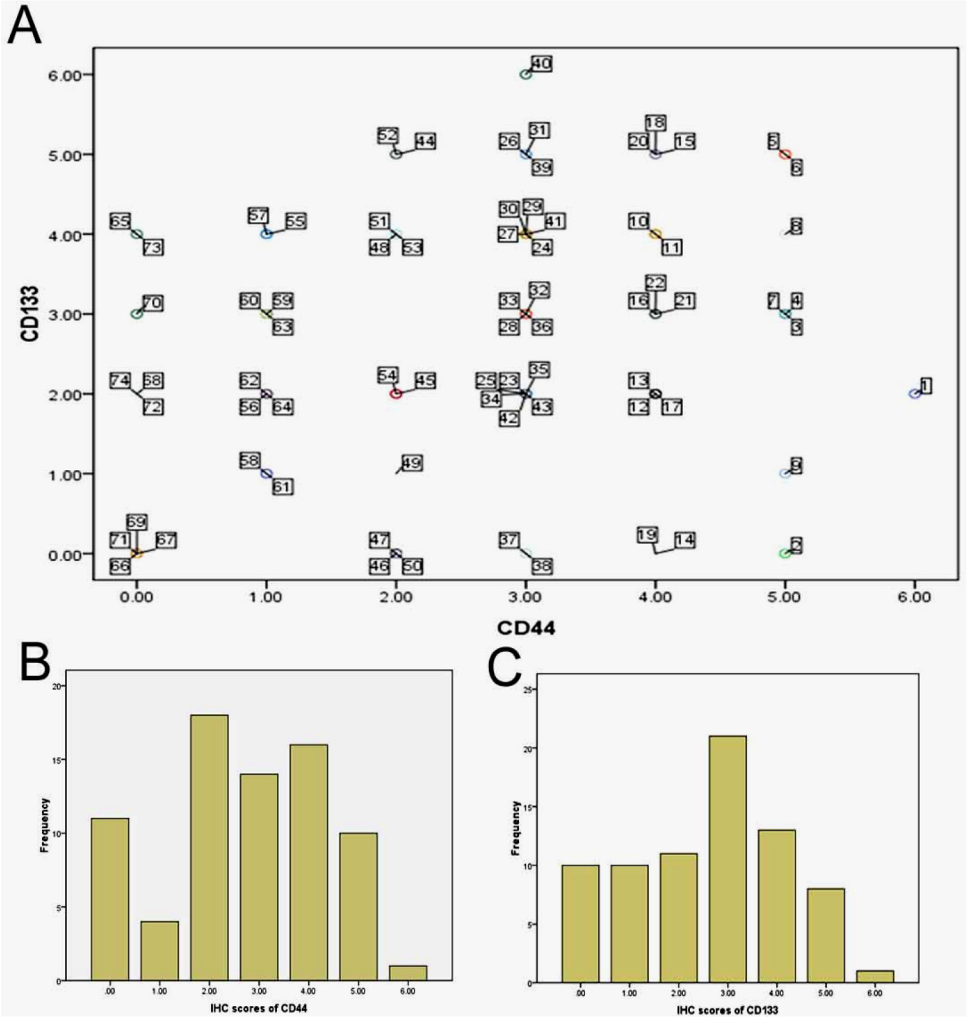

Supplementary Figure S2: Detailed quantification of samples in HCC patients stained by scoring system (A), and IHC scores of CD44 (B) and CD133 (C), respectively.

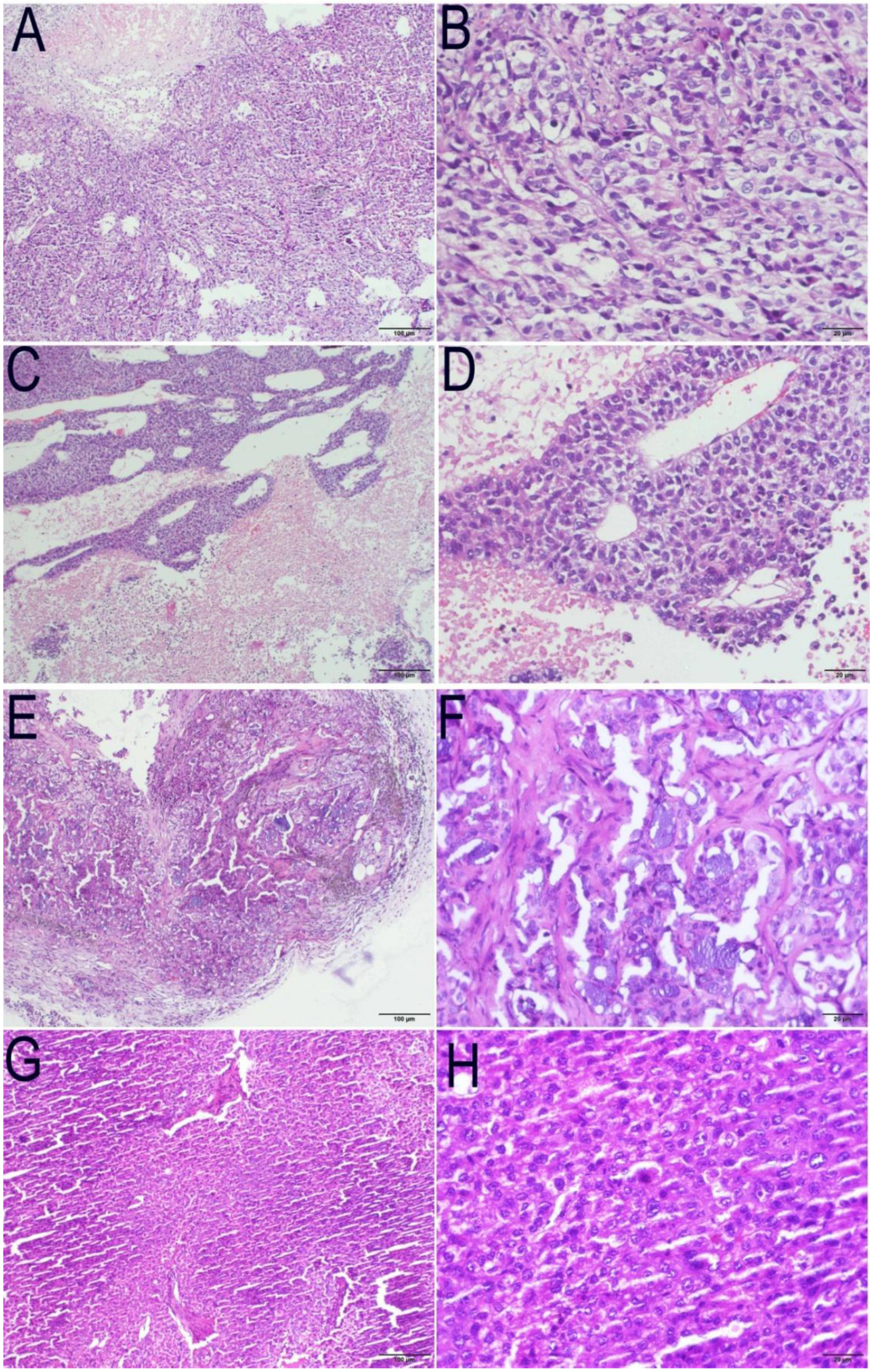

Supplementary Figure S3: Low magnification (left panel, 100×) and high magnification (right panel, 400×) using HE staining from the IHC staining cases of the PDX models. Representative case 1 HE staining (**A**–**B**) and its high expression of CD133 in Figure 5 vs case 2 HE staining (**C**–**D**) and its low expression of CD133 in Fig 5; Representative case 3 HE staining (**E**–**F**) and its high expression of CD44 in Fig 5 vs case 4 HE staining (**G**–**H**) and its low expression of CD44 in Figure 5 of the text.

Supplementary Table S1: The primer sequence of 12 CSC markers

Gene	Primers (5′→3′)	GenBank Accession
CD133	Fwd TGGATGCAGAACTTGACAACGT	NM_006017.2
	Rev ATACCTGCTACGACAGTCGTGGT	
CD90	Fwd GACCCGTGAGACAAAGAAGC	NM_006288.4
	Rev GCCCTCACACTTGACCAGTT	
CD44	Fwd ATGGACAAGTTTTGGTGGCACGC	NM_001202556.1
	Rev AAGATGTAACCTCCTGAAGTGCTGC	
ALDH1	Fwd ATCTTTGCTGACTGTGACCT	NM_012190.3
	Rev GCACCTCTTCTACCACTCTC	
Vimentin	Fwd GGGACCTCTACGAGGAGGAG	NM_003380.3
	Rev CGCATTGTCAACATCCTGTC	
Oct-4	Fwd GCAGCGACTATGCACAACGA	NM_002701
	Rev CCAGAGTGGTGACGGAGACA	
Sox-2	Fwd CATCACCCACAGCAAATGACA	NM_003106.2
	Rev GCTCCTACCGTACCACTAGAACTT	
CK 7	Fwd TGAATGATGAGATCAACTTCCTCAG	NM_005556.3
	Rev TGTCGGAGATCTGGGACTGC	
CK19	Fwd CGGGCATAAAAGGCGCCAGGT	NM_182920.1
	Rev GAGACCCCGAAGACGACAGGAAAC	
Nestin	Fwd TCGCTTAGAGGTGCAACAGC	NM_012987
	Rev CTCTGTAGACCTTGCTTCTCC	
CD13	Fwd ACGCCACCTCTACATCATC	NM_001150
	Rev AGCACCACCTCCTTGTTCTC	
EpCam	Fwd AGCTCAGGAAGAATGTG	NM_002354
	Rev TGAAGTACACTGGCATTGACG	
GAPDH	Fwd AATGAAGGGGTCATTGATGG	NM_002046
	Rev AAGGTGAAGGTCGGAGTCAA	

Supplementary Table S2: The IHC scores in CD44 high/low groups and CD133 high/low groups in PDX models

# of mice	Scores of CD44 expression in CD133 low group	# of mice	Scores of CD44 expression in CD133 high group	# of mice	Scores of CD133 expression in CD44 low group	# of mice	Scores of CD133 expression in CD44 high group
1	4	1	5	1	5	1	5
2	2	2	5	2	2	2	4
3	1	3	2	3	3	3	2
4	2	4	2	4	2	4	2
5	2	5	3	5	2	5	2
6	2	6	3	6	3	6	1
		7	5			7	3
		8	5			8	2
		9	4			9	3
		10	1			10	2
		11	2			11	5
		12	2			12	3
		13	4			13	1
		14	3			14	3
		15	2			15	3
		16	6			16	2
		17	2			17	2
		18	3			18	3
